# Systematic proteomics reveals plasma NEFL as a robust predictor and pathological associate in *C9ORF72*-related neurodegeneration

**DOI:** 10.3389/fnagi.2026.1792887

**Published:** 2026-04-21

**Authors:** Zhen Hu, Jing-jin Wan, Qin-qin Yan, Yu Fan, Jun Liu

**Affiliations:** 1 Department of Neurology, Ruijin Hospital Luwan Branch, Shanghai Jiao Tong University School of Medicine, Shanghai, China; 2 Department of Neurology and Institute of Neurology, Ruijin Hospital, Shanghai Jiao Tong University School of Medicine, Shanghai, China; 3 Department of Surgery, Renji Hospital, Shanghai Jiao Tong University School of Medicine, Shanghai, China; 4 Department of Radiology, Ruijin Hospital, Shanghai Jiao Tong University School of Medicine, Shanghai, China

**Keywords:** *C9ORF72*, motor neuron disease (MND), NEFL, neurodegeneration, repeat expansion

## Abstract

**Background:**

The *C9ORF72* repeat expansion is the most common genetic cause of amyotrophic lateral sclerosis (ALS) and frontotemporal dementia (FTD). While neurofilament light chain (NEFL) is an established biomarker of neuroaxonal damage, its specific dose-response relationship with the *C9ORF72* expansion and its potential role beyond a passive bystander require systematic investigation. We performed a proteome-wide screen to identify plasma proteins linked to the *C9ORF72* expansion and evaluated their predictive value for motor neuron disease (MND).

**Methods:**

We utilized whole-genome sequencing and plasma proteomics from the UK Biobank, analyzing 106 individuals with *C9ORF72* expansions (defined as >30 repeats) and 212 age- and sex-matched controls. We screened ~3,000 proteins for associations with the continuous repeat count. The top candidate was evaluated using restricted cubic splines (RCS) to assess non-linearity and threshold effects. Its ability to independently predict MND risk was tested using regression models and a machine learning approach.

**Results:**

Our unbiased screen identified NEFL as the sole protein significantly associated with the *C9ORF72* repeat count (FDR-adjusted *P* = 8.39 × 10^−4^). NEFL levels demonstrated a step-wise increase with expansion size, which followed a stable linear trajectory across the repeat spectrum (*P*_non − linear_ = 0.4435). Elevated NEFL independently predicted MND risk (OR = 2.42; HR = 2.90), even after adjusting for the *C9ORF72* repeat count. Our predictive model, combining NEFL and repeat count, achieved an AUC of 0.941 with 100% sensitivity. These findings align with emerging evidence that secreted NEFL may actively modulate neuroinflammation.

**Conclusions:**

NEFL emerges as a robust and specific plasma biomarker for *C9ORF72*-related neurodegeneration. Its strong linear association with repeat burden and independent predictive power, contextualized within its potential role in immune activation, suggest that NEFL is deeply integrated into the *C9ORF72* pathological landscape. These findings support NEFL-based screening and monitoring strategies for early intervention in *C9ORF72* carriers.

## Introduction

Amyotrophic lateral sclerosis (ALS) and frontotemporal dementia (FTD) are devastating neurodegenerative disorders that share significant clinical and pathological overlap (Rummens and Da Cruz, 2025; Hsiung et al., 2012). A major breakthrough in understanding these conditions came with the discovery of an intronic GGGGCC hexanucleotide repeat expansion in the *C9ORF72* gene, now recognized as the most common genetic cause of both familial and sporadic cases of ALS and FTD (DeJesus-Hernandez et al., 2011; Renton et al., 2011). This repeat expansion, through complex gain-of-function mechanisms—including the formation of toxic dipeptide repeat proteins and RNA foci—leads to widespread neurodegeneration (Ince et al., 2011; Cruts et al., 2013). While these genetic insights have been transformative, the precise sequence of pathogenic events remains poorly understood, and the identification of robust diagnostic and prognostic biomarkers remains an urgent clinical priority.

Recent advances have positioned neurofilament light chain (NEFL) as a premier biomarker at the crossroads of clinical diagnosis and the evaluation of gene-directed therapies (MC et al., 2024). Measurable in both cerebrospinal fluid and blood, NEFL levels reflect neuroaxonal damage and correlate strongly with disease severity and progression across various neurodegenerative conditions, including ALS and FTD (MC et al., 2024; Davydow et al., 2026). However, emerging evidence suggests that NEFL may be more than a passive indicator of neuronal injury; secreted NEFL may actively induce myeloid cell activation and neuroinflammation, thereby potentially contributing to the neurodegenerative process (Kahn et al., 2025).

The advent of large-scale, population-based biobanks and advanced proteomic technologies offers an unprecedented opportunity to discover such pivotal biomarkers (Deng et al., 2025; Guo et al., 2024). By systematically screening the plasma proteome of individuals with a known genetic risk factor, we can identify proteins whose expression is directly influenced by the underlying genetic pathology. This unbiased, discovery-driven approach holds the potential to reveal biomarkers that are not merely markers of generalized neuronal injury, but are intrinsically linked to the specific pathogenic cascade initiated by the genetic mutation.

In this study, we leveraged the unparalleled scale of the UK Biobank, combining comprehensive whole-genome sequencing (WGS) with plasma proteomics data from a large population cohort. Our primary objective was to perform a systematic and unbiased screen of nearly 3,000 plasma proteins to identify those associated with the *C9ORF72* repeat expansion count. Following this initial screen, we focused on the most promising candidate—NEFL—to conduct a more detailed investigation. We hypothesized that NEFL would demonstrate a clear dose-dependent relationship with repeat count and serve as an independent predictor of MND risk, reflecting its significant role in the *C9ORF72* pathological landscape. We further aimed to develop a robust machine learning model to assess the clinical utility of NEFL as a screening and diagnostic biomarker for *C9ORF72*-related neurodegeneration.

## Methods

### Ethical considerations

This study was conducted under UK Biobank (Bycroft et al., 2018) application number 162635. The UK Biobank has received ethical approval from the North West Multi-Centre Research Ethics Committee (https://www.ukbiobank.ac.uk/learn-more-about-uk-biobank/about-us/ethics), and all participants provided informed consent. No additional ethical approval was required for this project.

### Study population and data curation

We utilized WGS and plasma proteomics data from the UK Biobank Research Analysis Platform (RAP). The WGS data (Data-Field 24048), processed with BWA-mem (Jung and Han, 2022), GATK pipelines (Ulintz et al., 2019) and DRAGEN 3.7.8 (Li, 2024; Behera et al., 2025), were used to determine *C9ORF72* GGGGCC repeat sizes. The plasma proteomics data (Data-Field 30900) were obtained from the UK Biobank Pharma Proteomics Project (UKB-PPP), which used the Olink platform to provide quantitative data for approximately 3,000 proteins from over 54,000 participants (Sun et al., 2022). Our study was restricted to participants of European ancestry.

As the pathological repeat-length threshold for *C9ORF72* has not been definitively established, we primarily utilized an arbitrary cutoff of >30 repeats to define cases, which is common practice in large-scale genetic studies (Balendra and Isaacs, 2018). To mitigate the potential bias of an arbitrary threshold, we primarily employed continuous repeat modeling in our discovery screen, treating the expansion size as a continuous variable to capture the biological gradient of the pathology (Balendra and Isaacs, 2018). To control for demographic confounders, we performed a 1:2 propensity score matching analysis. From a pool of 41,933 individuals, we selected controls with a repeat count of ≤ 10. Using the R package *MatchIt*, we matched cases to controls based on age and sex. The final analytical cohort consisted of 318 individuals (106 cases, 212 controls).

### Bioinformatic and statistical analysis

#### C9ORF72 repeat expansion analysis

We quantified the size of the *C9ORF72* repeat expansion using ExpansionHunter (version 4.0.2; Dolzhenko et al., 2019). The maximum repeat size reported was used as our primary measure of expansion length.

### Proteome-wide association screen and threshold effect evaluation

We conducted an unbiased proteome-wide screen using linear regression to model the association between each protein and the continuous repeat count, adjusting for age, sex, and the first 10 principal components of ancestry. We applied a false discovery rate (FDR) correction for multiple testing. To further justify our threshold and evaluate potential non-linear or “threshold” effects as suggested by previous literature (Balendra and Isaacs, 2018), we employed restricted cubic splines (RCS) with four knots. This approach allowed us to visualize the dose-response relationship across the entire spectrum of repeat counts without relying solely on a fixed binary cutoff.

### Sub-group analysis and dose-effect relationship

Analysis of variance (ANOVA) was performed to compare NEFL expression across predefined repeat count groups (Control, G30_60, G60_200, G200_600, G600_1000, and G1000_up), followed by *post-hoc* Tukey tests.

### Risk prediction models

Our MND cohort was defined using the UK Biobank's algorithmically-derived outcomes. To determine if NEFL independently predicts MND risk, we performed logistic regression and Cox proportional hazards modeling, adjusting for age, sex, ancestry, and the continuous *C9ORF72* repeat count.

### Machine learning model

To evaluate clinical utility, a random forest model was trained on a 70% subset and validated on a 30% held-out test set. Model performance was evaluated using the Area Under the Curve (AUC) with 95% confidence intervals, balanced accuracy, sensitivity, and specificity. Variable importance plots were generated to identify the most influential predictors.

### Statistical analysis and software

All statistical analyses were performed using R (version 4.2.1) with packages including *tidyverse, rms, MatchIt, survival, survminer, broom, caret, pROC*, and *randomForest*. *P*-values below 0.05 were considered statistically significant.

## Results

### Cohort characteristics and patient-control matching

In our study, we utilized WGS data from over 500,000 UK Biobank participants to determine the *C9ORF72* GGGGCC repeat size. Among these, plasma proteomics data (median 2,894 proteins measured) were available for 53,058 individuals. Following primary filtering, our cohort comprised 42,039 Caucasian individuals with both genetic and proteomic data. We identified 106 individuals with more than 30 GGGGCC repeats, which we designated as cases. To mitigate potential confounding by age and sex, we performed 1:2 propensity score matching, selecting 212 controls with ≤ 10 repeats from a pool of 41,933 individuals.

**Figure 1:**
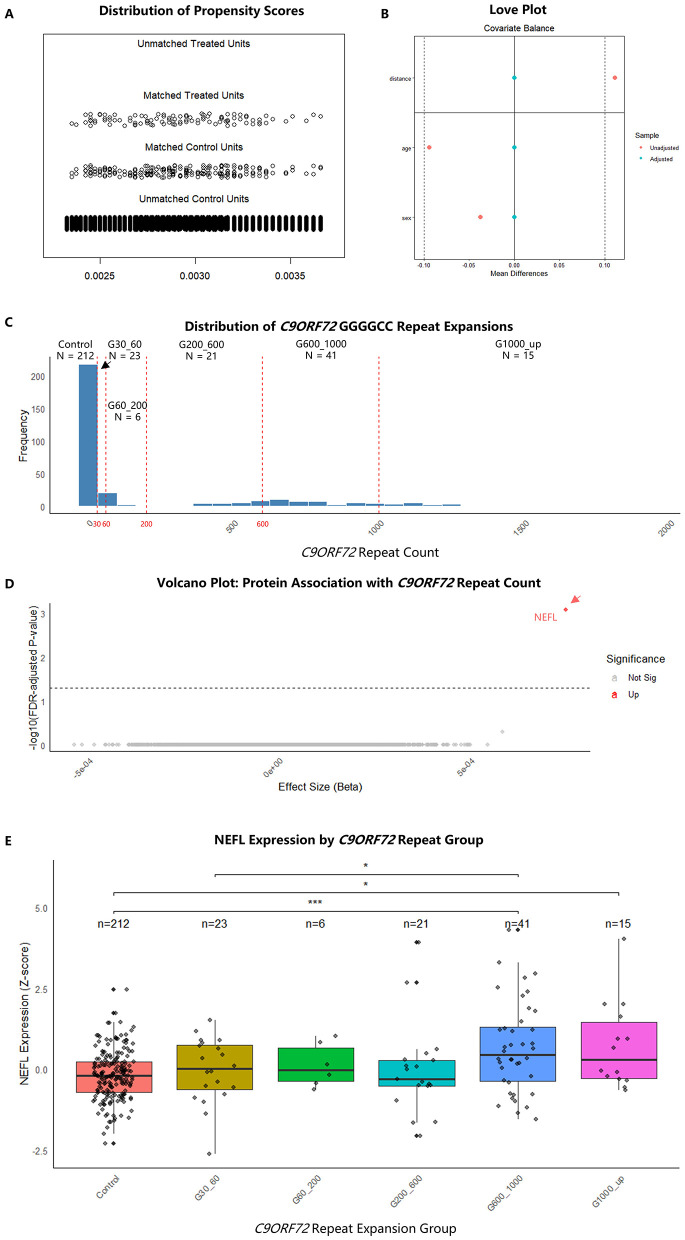
Cohort construction and identification of NEFL as a specific biomarker for *C9ORF72* repeat expansion. **(A)** Distribution of propensity scores before and after 1:2 propensity score matching. **(B)** Love plot displaying the standardized mean differences for age and sex before and after propensity score matching, demonstrating successful balancing of the case and control cohorts. **(C)** Distribution of *C9ORF72* GGGGCC repeat expansion sizes in the final analytical cohort (*n* = 318). Dashed red lines indicate the thresholds used for group stratification. Bar plot showing the number of individuals in each pre-defined *C9ORF72* repeat expansion group: control (≤10 repeats, *n* = 212), G30_60 (*n* = 23), G60_200 (*n* = 6), G200_600 (*n* = 21), G600_1000 (*n* = 41), and G1000_up (>1,000 repeats, *n* = 15). **(D)** Volcano plot of the proteome-wide association analysis. Each point represents one of 2,923 plasma proteins. Associations were modeled using linear regression with repeat count as a continuous variable, adjusted for age, sex, and genetic principal components. The dashed horizontal line indicates the threshold for significance after False Discovery Rate (FDR) correction (adjusted *P* < 0.05). Neurofilament light chain (NEFL) was the sole protein surpassing this threshold. **(E)** Boxplot of plasma NEFL levels (*Z*-score) across *C9ORF72* repeat expansion groups. Boxes represent the interquartile range (IQR), the horizontal line is the median, and whiskers extend to 1.5 IQR. Individual data points are shown. ^*^P value < 0.05, ^***^P value < 0.001.

This matching process successfully balanced the cohorts. The matched groups were identical in terms of age and sex distribution (Figures 1A, B). The final analytical cohort consisted of 318 individuals, with a detailed breakdown by repeat group presented in Figure 1C: Control (*n* = 212), G30_60 (*n* = 23), G60_200 (*n* = 6), G200_600 (*n* = 21), G600_1000 (*n* = 41), and G1000_up (*n* = 15).

### NEFL as the sole specific plasma biomarker for *C9ORF72* repeat expansion

We conducted a comprehensive screen of nearly 3,000 plasma proteins to identify those associated with the *C9ORF72* repeat expansion count. After controlling for age, sex, and the first 10 principal components of ancestry, NEFL emerged as the only protein with a significant association after stringent FDR correction (FDR adjusted *P* = 8.39 × 10^−4^, Supplementary Table 1). NEFL expression showed a significant association with *C9ORF72* repeat count (estimate = 7.55 × 10^−4^, *P* = 2.87 × 10^−7^). The volcano plot in Figure 1D highlights NEFL as the standout hit from our proteomic screen.

### Dose-effect relationship and threshold evaluation

ANOVA across the different repeat count groups confirmed that NEFL demonstrated a significant difference in expression (FDR adjusted *P* < 0.05, Supplementary Table 2). Subsequent Tukey *post-hoc* tests revealed a step-wise increase in NEFL levels, which was most pronounced in individuals with larger expansions. Specifically, NEFL expression was significantly higher in the G600_1000 group (adjusted *P* = 1.40 × 10^−5^) and the G1000_up group (adjusted *P* = 1.09 × 10^−2^) compared to controls (Supplementary Table 3, Figure 1E).

**Figure 2:**
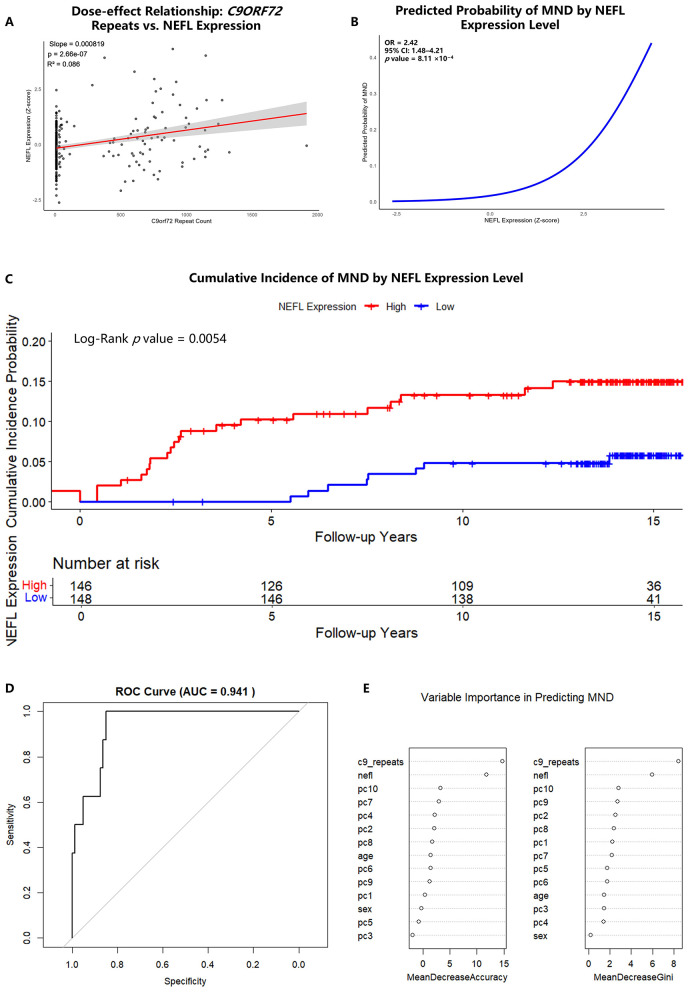
NEFL predicts motor neuron disease risk independently of genetic burden and enables high-accuracy prediction. **(A)** Scatter plot showing the dose-effect relationship between continuous *C9ORF72* repeat count and plasma NEFL level (*Z*-score). The solid red line represents the linear regression fit, with the shaded area indicating the 95% confidence interval. This linear association was further validated by restricted cubic spline analysis (see Supplementary Figure 1). The regression equation, slope, and *P*-value are annotated. **(B)** Predicted probability of motor neuron disease (MND) as a function of plasma NEFL level from the multivariable logistic regression model. The curve shows the probability while holding all other covariates (age, sex, *C9ORF72* repeat count, genetic principal components) at their mean values. The odds ratio (OR) and *P*-value for NEFL from the full model are annotated. **(C)** Cumulative incidence curves for MND onset stratified by plasma NEFL level (high vs. low, based on a median split). The *P*-value from the Log-rank test is shown. **(D)** Receiver Operating Characteristic (ROC) curve demonstrating the performance of the random forest machine learning model in predicting MND on the held-out test set (30% of data). The area under the curve (AUC) is shown with its 95% confidence interval [0.884–0.998]. **(E)** Variable importance plot from the random forest model. Features are ranked by their mean decrease in Gini impurity. NEFL and *C9ORF72* repeat count emerged as the two most influential predictors for MND risk within the *C9ORF72* expansion landscape.

To further evaluate the nature of this dose-effect relationship and explore potential non-linear or threshold effects, we performed RCS analysis. While a visual trend of accelerated NEFL increase was noted at higher repeat counts, the formal test for non-linearity was non-significant (*P*_non − linear_ = 0.4435, Supplementary Figure 1). This suggests that a linear model provides a statistically robust representation of the association between repeat expansion size and NEFL levels in this cohort. A scatter plot further visualized this correlation (Slope = 8.19 × 10^−4^, *P* = 2.66 × 10^−7^, R^2^ = 0.086; Figure 2A).

### Elevated NEFL independently predicts MND risk

To explore NEFL's potential as an independent risk predictor for MND, we performed logistic regression and Cox proportional hazards modeling, adjusted for age, sex, ancestry, and the continuous *C9ORF72* repeat count.

The logistic regression model revealed that for every one standard deviation increase in NEFL expression, the odds of having MND increased by 2.42-fold (OR = 2.42, 95% CI: 1.48–4.21; *P* = 8.11 × 10^−4^, Supplementary Table 4). The predictive curve in Figure 2B illustrates this relationship. Consistent with this, the Cox model showed that elevated NEFL was associated with a 2.90-fold increased risk of developing MND (HR = 2.90, 95% CI: 1.96–4.28; *P* = 9.50 × 10^−8^, Supplementary Table 5), even after accounting for the *C9ORF72* repeat count. The cumulative incidence plot demonstrated a significantly higher incidence of MND in the high-NEFL group over a 15-year follow-up period (Log–Rank *P* = 0.0054; Figure 2C).

### Clinical utility of NEFL and repeat count in MND prediction

We developed a random forest machine learning model to predict MND risk. The model demonstrated robust predictive power, achieving an AUC of 0.941 (95% CI: 0.884–0.998; Figure 2D), indicating strong discrimination. On the held-out test set, the model yielded a balanced accuracy of 0.925 (Supplementary Table 6). Notably, the model correctly identified all true MND cases (Sensitivity = 100%), with a specificity of 85.0%, a Positive Predictive Value (PPV) of 36.4%, and a Negative Predictive Value (NPV) of 100%.

Analysis of feature importance confirmed that NEFL expression and *C9ORF72* repeat count were the most influential predictors, surpassing all other variables (Figure 2E). These findings provide evidence for the close association of NEFL with the pathological progression of *C9ORF72*-related neurodegeneration.

## Discussion

In this study, we leveraged the vast scale and multimodal data of the UK Biobank to provide novel insights into the relationship between *C9ORF72* repeat expansions and plasma NEFL, establishing NEFL as a robust and independent biomarker for MND. Our findings advance the field by demonstrating a clear dose-effect relationship, uncovering the deep integration of NEFL within the *C9ORF72* pathological landscape, and validating its exceptional predictive power in a large-scale, population-based cohort.

Our discovery that NEFL is the sole plasma protein significantly associated with *C9ORF72* repeat expansion among thousands of candidates is a powerful and specific finding. While previous studies have noted associations between NEFL and *C9ORF72* carriers (Thomas et al., 2025; Verde, 2025; Huang et al., 2020; Saracino et al., 2021), these were largely confined to smaller, clinically-ascertained cohorts. Our population-based approach provides definitive evidence for this link, minimizing selection bias. The demonstration of a dose-dependent, step-wise increase in NEFL levels with escalating repeat counts offers a critical layer of insight. Notably, our RCS analysis yielded a non-significant non-linearity test (*P*_non − linear_ = 0.4435), suggesting that the relationship between repeat burden and neuroaxonal damage—as indexed by NEFL—follows a stable linear trajectory across the expansion spectrum in this cohort. This consistent dose-response provides a quantifiable measure of disease burden that may inform clinical stratification and the monitoring of gene-directed therapies (MC et al., 2024).

A central finding of our study is that elevated NEFL independently predicts MND risk, even after accounting for the *C9ORF72* repeat count. This challenges the “bystander” hypothesis (Thomas et al., 2025; Verde, 2025), which posits that NEFL is merely a passive indicator of neuronal damage. Instead, our results support a model where NEFL is an active participant in the pathological cascade. Emerging evidence indicates that secreted NEFL following neuronal injury is not biologically inert; it can trigger calpain-driven proteolysis and subsequently activate myeloid cells and microglia, thereby driving neuroinflammation and accelerating disease progression (Kahn et al., 2025). Thus, the *C9ORF72* repeat expansion may initiate a self-reinforcing loop where initial axonal damage leads to NEFL release, which in turn exacerbates neurodegeneration through inflammatory pathways.

The clinical utility of our findings is underscored by the exceptional performance of our machine learning model (AUC = 0.941). With 100% sensitivity and a NPV of 100% on the held-out test set, the model demonstrates that the combination of NEFL and *C9ORF72* repeat count can serve as a highly effective screening tool to exclude non-cases in a general population setting. This is particularly relevant for differentiating neurodegenerative conditions like behavioral variant FTD (bvFTD) from primary psychiatric disorders, where NEFL has shown high diagnostic accuracy (Davydow et al., 2026). In the context of emerging therapies, such as Tofersen for *SOD1*-ALS, using NEFL as a surrogate pharmacodynamic marker has gained regulatory acceptance (MC et al., 2024); our results suggest a similar potential for *C9ORF72*-targeted interventions.

While our study offers significant contributions, certain limitations warrant consideration. The number of individuals with *C9ORF72*-associated MND in the UK Biobank remains small (UK Biobank Whole-Genome Sequencing Consortium, 2025). The retrospective nature limits our ability to track the longitudinal trajectory of NEFL leading up to onset. Additionally, while we have established a strong association, further functional studies are required to fully elucidate the inflammatory mechanisms of extracellular NEFL.

The robustness of our findings is underpinned by a rigorous methodological approach. We utilized WGS data, which has demonstrated high diagnostic accuracy compared to the gold-standard PCR testing (Ibañez et al., 2022). However, we acknowledge the inherent limitations of short-read sequencing in capturing the full structural complexity of long repeat expansions. Future studies utilizing long-read sequencing technologies, such as Oxford Nanopore or PacBio, will be valuable to provide a more definitive characterization.

In conclusion, our study provides compelling evidence that plasma NEFL is a specific and quantitative biomarker for *C9ORF72* repeat expansions. By demonstrating its dose-effect relationship, independent predictive power, and diagnostic accuracy, we have positioned NEFL as a central player in the *C9ORF72* pathological process. This work paves the way for NEFL-based screening strategies and opens new avenues for therapeutic interventions targeting the neuroinflammatory pathways in *C9ORF72*-related neurodegeneration.

## Data Availability

Data used in this study are available from the UK Biobank (accession number 162635) through the UK Biobank Access Management System (https://www.ukbiobank.ac.uk/). Plasma proteomics data were obtained from the UK Biobank Pharma Proteomics Project (Olink platform). Derived data fields generated in this study will be returned to the UK Biobank in accordance with their data sharing policies.
