# Supplementary material for: Systematic proteomics reveals plasma NEFL as a robust predictor and pathological associate in C9ORF72-related neurodegeneration
Source: Front Aging Neurosci. 2026 Apr 21;18:1792887. doi: 10.3389/fnagi.2026.1792887 (PMC13139100; doi:10.3389/fnagi.2026.1792887)
Supplement: Supplementary file 2 [file Data_Sheet_1.docx]

**Supplementary Materials**

Supplementary Figure 1 Restricted Cubic Spline (RCS) Analysis of the Association Between NEFL and *C9ORF72* Repeat Count


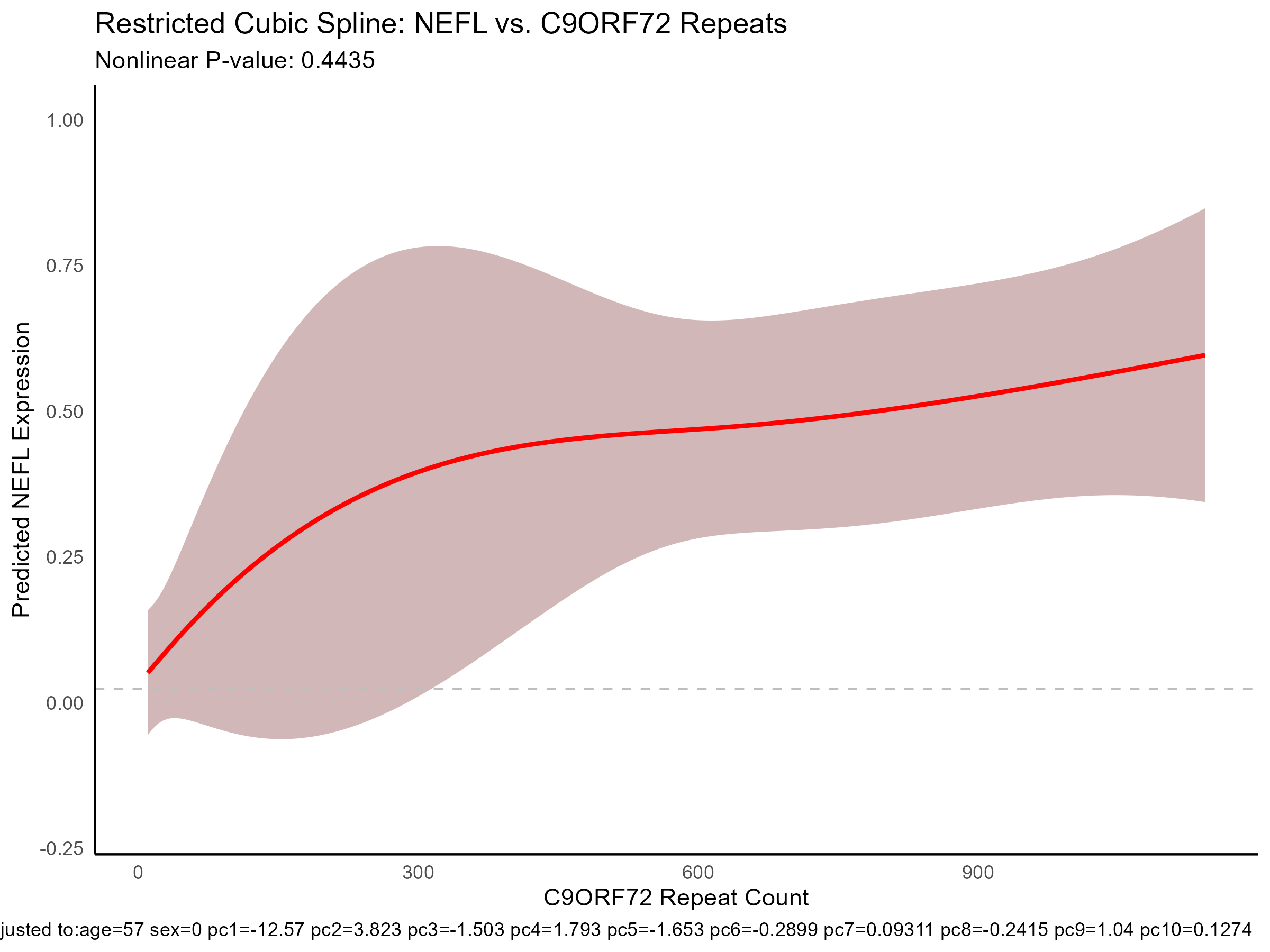


The solid red line represents the predicted NEFL expression levels modeled using a restricted cubic spline (RCS) with four knots, adjusted for age, sex, and the first 10 principal components of ancestry. The shaded light-red area denotes the 95% confidence interval (CI). The horizontal dashed grey line indicates the median NEFL expression level of the control group (repeat count ≤ 10). While a stepwise increase in NEFL is observed with increasing repeat counts, the formal test for non-linearity was non-significant (*P*_non-linear_ = 0.4435), suggesting that a linear model adequately captures the dose-response relationship in the current cohort. The apparent fluctuations in the curve at higher repeat counts (e.g., >600) likely reflect increased variance due to the sparse sample size in extreme expansion ranges.
